# Cuticle thickening associated with pyrethroid resistance in the major malaria vector *Anopheles funestus*

**DOI:** 10.1186/1756-3305-3-67

**Published:** 2010-08-04

**Authors:** OR Wood, S Hanrahan, M Coetzee, LL Koekemoer, BD Brooke

**Affiliations:** 1 Malaria Entomology Research Unit, School of Pathology of the University of the Witwatersrand and the National Health Laboratory Service, Johannesburg, South Africa; 2 Vector Control Reference Unit, National Institute for Communicable Diseases, NHLS, Private Bag X4, Sandringham, 2131, South Africa; 3 School of Animal, Plant and Environmental Sciences, University of Witwatersrand, Johannesburg, South Africa

## Abstract

**Background:**

Malaria in South Africa is primarily transmitted by *Anopheles funestus *Giles. Resistance to pyrethroid insecticides in *An. funestus *in northern Kwazulu/Natal, South Africa, and in neighbouring areas of southern Mozambique enabled populations of this species to increase their ranges into areas where pyrethroids were being exclusively used for malaria control. Pyrethroid resistance in southern African *An. funestus *is primarily conferred by monooxygenase enzyme metabolism. However, selection for this resistance mechanism is likely to have occurred in conjunction with other factors that improve production of the resistance phenotype. A strong candidate is cuticle thickening. This is because thicker cuticles lead to slower rates of insecticide absorption, which is likely to increase the efficiency of metabolic detoxification.

**Results:**

Measures of mean cuticle thickness in laboratory samples of female *An. funestus *were obtained using scanning electron microscopy (SEM). These females were drawn from a laboratory colony carrying the pyrethroid resistance phenotype at a stable rate, but not fixed. Prior to cuticle thickness measurements, these samples were characterised as either more or less tolerant to permethrin exposure in one experiment, and either permethrin resistant or susceptible in another experiment. There was a significant and positive correlation between mean cuticle thickness and time to knock down during exposure to permethrin. Mean cuticle thickness was significantly greater in those samples characterised either as more tolerant or resistant to permethrin exposure compared to those characterised as either less tolerant or permethrin susceptible. Further, insecticide susceptible female *An. funestus *have thicker cuticles than their male counterparts.

**Conclusion:**

Pyrethroid tolerant or resistant *An. funestus *females are likely to have thicker cuticles than less tolerant or susceptible females, and females generally have thicker cuticles than males. In pyrethroid resistant *An. funestus*, this increase in cuticle thickness is likely to have developed as an auxiliary to the primary mode of pyrethroid resistance which is based on enzyme-mediated detoxification.

## Background

Malaria in southern Africa is transmitted by *Anopheles funestus *Giles, the nominal member of the *An. funestus *species group, and *An. arabiensis *Patton, a member of the *An. gambiae *species complex.

Malaria vector control in South Africa and many of its neighbouring countries is based on the application of insecticides inside human dwellings and other structures in affected areas. The effectiveness of this approach hinges on production issues such as insecticide formulation, logistical issues such as adequate and timely coverage, and biological issues of which the emergence of insecticide resistance in target mosquito populations is the most pressing.

Resistance to pyrethroid insecticides in *An. funestus *in northern Kwazulu/Natal, South Africa, and in neighbouring areas of southern Mozambique enabled populations of this species to increase their ranges into areas where pyrethroids were being exclusively used for malaria control [1-3]. The effect of this range expansion, as well as the emergence of anti-malarial drug resistance, was an unprecedented malaria epidemic, primarily in Kwazulu/Natal, South Africa, during the period 1996 to 2000. Adequate responses including the re-introduction of DDT and a change in the prescribed anti-malarial drug regimen succeeded in controlling the epidemic [3,4]. Nevertheless, this incident highlighted the potential effect that insecticide resistance can have on an otherwise well-implemented vector control programme.

Pyrethroid resistance in southern African *An. funestus *is primarily based on an enzyme system that employs the up-regulated detoxifying capabilities of at least two P450 monooxygenase genes [2,5-7], with variation in expression of the resistance phenotype by age and gender [8]. It is likely that resistance in *An. funestus *arose comparatively rapidly as a consequence of intense insecticide selection pressure [1]. As a result, other factors may be directly or indirectly involved in the expression of the resistance phenotype. One possibility is cuticular thickening. Thicker cuticles lead to slower rates of insecticide absorption, which is likely to enhance the efficiency of metabolic detoxification. Slower insecticide penetration across the cuticle (though not necessarily the result of cuticle thickening) has been associated with insecticide resistance in the cotton bollworm *Helicoverpa armigera *[9,10].

Cuticle thickening is a gene-regulated process that an insect undergoes as it ages. It has been demonstrated that cuticle is laid down in a circadian pattern, resulting in growth rings of nocturnal lamellate and diurnal non-lamellate structure [11-13]. The periods of transcription of genes coding for cuticle formation have been used to predict age in the dengue vector *Aedes aegypti *in the field [14], and aging by quantification of cuticle rings has been described for African migratory locusts [15].

Measurable cuticle thickening has been associated with pyrethroid resistance in the Chagas disease vector *Triatoma infestans *[16] and is tentatively inferred from micro-array gene transcription analysis in *An. stephensi *[17]. Measurements of rates of insecticide penetration have been found to be affected by thickened cuticles as well as by other structural components of cuticles such as relative amounts of surface hydrocarbons [16], suggesting that decreased rates of penetration across the cuticle slows insecticide inoculation of internal organs sufficiently to allow for effective metabolically-mediated detoxification.

In a preliminary micro-array analysis of pyrethroid resistant and susceptible *An. funestus *[unpublished data] using the *An. gambiae *detoxification chip [18], differential transcription of a gene associated with cuticle deposition (JV2) was observed. This provided the impetus to compare cuticle thicknesses between laboratory reared *An. funestus *samples characterised by their responses to pyrethroid intoxication. A direct measure of association between these two phenotypes (response to insecticide intoxication and cuticle thickness) provides a critical platform for establishing whether they share a causal relationship, as well as for predicting the potential biological and epidemiological implications of such a relationship.

## Methods

### Permethrin tolerance assay

#### Anopheles funestus samples

Samples of *An. funestus *were drawn from the FUMOZ laboratory colony housed at the VCRU, NICD, in Johannesburg. This colony originates from material collected in southern Mozambique and is maintained under standard insectary conditions [8]. FUMOZ carries resistance to the pyrethroid insecticide permethrin at comparatively stable rates ranging between 5% and 30% mortalities [19], as measured using standard insecticide exposure assays against adults [20]. For the gender comparison, samples of *An. funestus *were drawn from the FANG laboratory colony. This colony originates from material collected in southern Angola and is fully susceptible to insecticides.

#### Insecticide exposure assay

In order to choose an appropriate adult mosquito age for cuticle thickness measurements, the findings of Cook *et al*. [14] concerning the expression periods of cuticle gene orthologue Ae-8505 in *Aedes aegypti *were used as a guide. It was decided to use ten day old female mosquitoes all drawn from the same FUMOZ cohort. This age cohort was chosen in an effort to minimise variation in thickness due to age dependent gene expression (allowing for variation of approximately 24 h as mosquito samples were collected once per day) as well as to obtain cuticles at their thickest in terms of growth and repair. It has previously been established that FUMOZ females show appreciable levels of pyrethroid resistance at 10 days [8].

Twenty adult females were aspirated into a WHO exposure tube containing a 0.75% permethrin treated filter paper supplied by the World Health Organization (WHO). Knockdown of mosquitoes in the exposure tube was continuously monitored for one hour. In order to validate knockdown of an individual, the base directly under the mosquito was lightly tapped in order to ascertain whether it was a true knockdown. Any individual that was still able to fly was kept in the exposure tube until it became completely moribund, at which point it was removed using an aspirator carefully inserted into the tube. The time of removal was recorded. Those individuals that were still active after 60 minutes exposure were grouped as + 60 minutes. These samples were cold-terminated in a fridge at 4°C within five minutes of removal from the permethrin exposure tube, preventing further cuticle formation in any of the mosquitoes taken for subsequent analysis. Females representing the earliest knock-down cohort were grouped and termed as permethrin intolerant (< 30 minutes to knock-down), whilst females with a knock-down time in excess of 40 minutes were grouped as permethrin tolerant. These time discriminators presented as natural limits for FUMOZ based on exposure observations and are not standardised for anophelines in general. This process was repeated until suitable samples (at least 10) of individuals from the intolerant and tolerant groupings had been collected.

#### Specimen preparation for SEM

**Figure 1:**
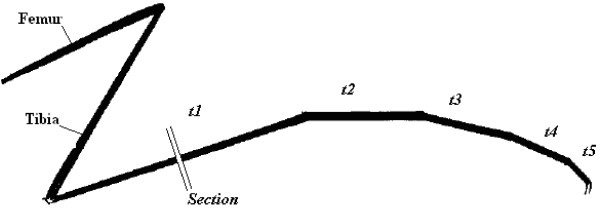
**Illustration of the point of section on *An. funestus *tarsomere 1 (after Evans **[24]**(*t1-t5 *= five tarsal segments)**.

**Figure 2:**
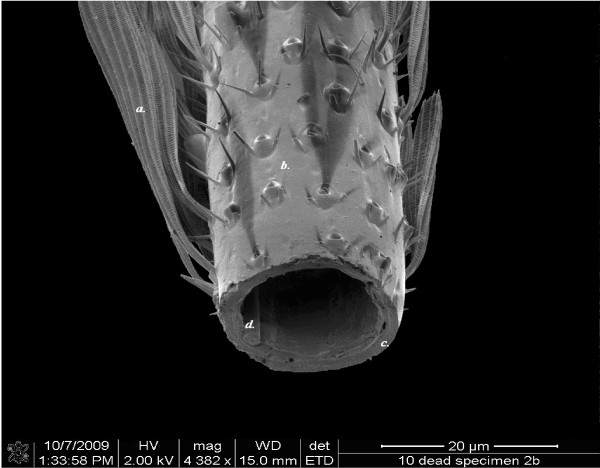
**SEM (FEI Quanta 400 E) micrograph showing an oblique view of the lower half of a sectioned leg**. a. Scale. b. Area where scales were brushed away to facilitate a clean section. c. The integument. d. Muscle or tendon (frequently used as a reference point for standardizing points of measure).

All females were washed twice in 70% ethanol in order to clean them thoroughly. The legs were given a light brushing in the area of the desired section while under the ethanol. This was done to remove some of the scales that cover the legs in order to facilitate a cleaner cut. Tarsomere I on the left middle leg was severed at the midpoint in a drop of alcohol using a new platinum coated blade (Figures 1 &2). Following the sectioning, the leg was washed again to remove any debris which may obscure the view of the cuticle. Fresh ethanol was gently passed over the section to remove any debris. The leg was left attached to the body. Each mosquito was then placed ventral side up in a foam critical point drying container with the sectioned portion of the leg orientated vertically. The containers were then passed through 70%, 80%, 90% and 100% ethanol. Each step lasted at least two hours and was repeated twice. The final change was allowed to stand overnight, and the ethanol used was kept under a molecular sieve to ensure complete dehydration. The dried specimens were mounted ventral side up on stubs, ensuring vertical positioning of the sectioned part of the leg so as to enable a square measurement across the section from above.

**Figure 3:**
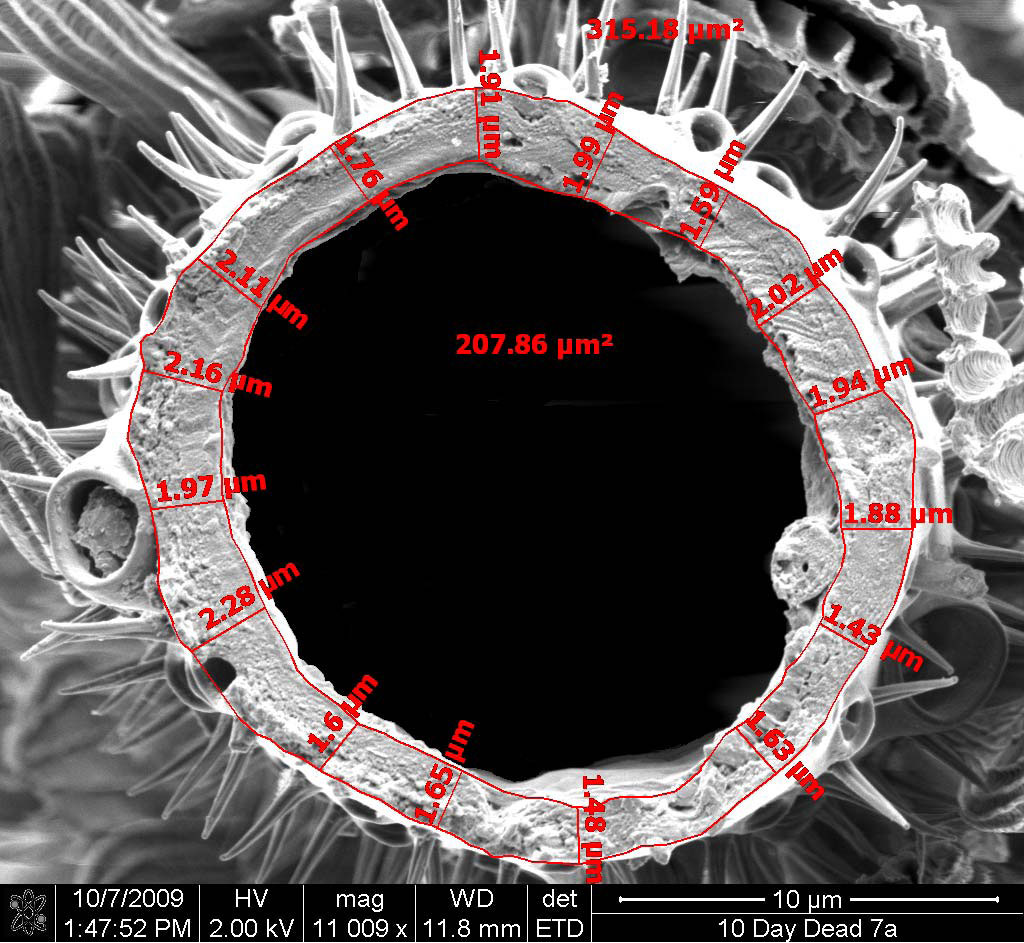
**Micrograph from the FEI Quanta 400E illustrating 16 initial points of cuticle thickness measurement, allowing for calculation of mean cuticle thickness per individual**. At least 25 points of measurements were used per individual.

Specimens were sputter coated with carbon and gold palladium and viewed using a Jeol JSM-840 scanning electron microscope. Micrographs obtained were digitized and examined using Zeiss AxioVision Release 4.6 software to measure the thickness of the cuticle (Figure 3). Measurements were made by tracing the outline of both the inner and outer circumference of the cuticle and measuring the shortest distance between the two at no fewer than 25 different, evenly distributed points with obvious aberrations such as scale beds excluded. A mean cuticle thickness per specimen was obtained in this way. The permethrin tolerant and intolerant groups were then compared using one-way ANOVA and a possible trend between cuticle thickness and time to knock-down was evaluated using linear regression (Statistix 7.0 software).

### Permethrin susceptibility assay

Following the tolerance assays, another large sample of FUMOZ females were taken from a single cohort. In order to offset the effect of circadian cuticular lay-down the sample population was aged to nine days at which point a sub-sample was removed for insecticide exposure while the remainder were allowed to age one more day. The nine and ten day old groups were exposed to 0.75% permethrin for 1 hour according to the standard WHO protocol for testing adult anopheline susceptibility to insecticide [20]. Exposures of both sub-samples were conducted at the same time of day under the same conditions.

Those survivors from the day 9 exposure were removed on the 10^th ^day following a 24 hour recovery period and were cold terminated as previously described. These survivors were characterised as 10-day-old permethrin resistant. Those females that succumbed to permethrin exposure during the day 10 exposure were characterised as 10-day-old permethrin susceptible.

In order to accommodate an effect of body size on cuticle thickness, the wing lengths of all females used in the permethrin resistance assay were measured. Wing length gives a good approximation of body size [21].

Specimen preparation for SEM was as described earlier. An FEI Quanta 400 E scanning electron microscope, which became available for use in the interim, was used to obtain digital micrographs. Mean cuticle thickness was compared between the permethrin resistant and susceptible samples using one-way ANOVA (Statistix 7.0 software).

### Gender comparison

Given the variation in insecticide resistance phenotypic expression between males and females in southern African *An. funestus *(females are generally more tolerant/resistant [8]), it was decided to ascertain whether there is significant variation in cuticle thickness by gender. A sample of males and females was removed from a cohort of the FANG colony. This colony was used for this experiment because it does not carry any measurable insecticide resistance phenotypes, thus removing insecticide resistance as a confounding variable when comparing cuticle thickness between males and females. The sample was aged to five days before being cold terminated as in the previous assays. The wing lengths of all individuals used for subsequent cuticle measurements were determined.

All specimens were prepared for SEM as described earlier, and the FEI Quanta 400 E scanning electron microscope was used to obtain digital micrographs. The images on all micrographs were analysed and measured as described earlier and the mean cuticle thickness of each specimen was calculated. Mean cuticle thickness was compared between males and females using one-way ANOVA (Statistix 7.0 software).

## Results

### Permethrin tolerance assay

During viewing it was noted that a number of the prepared specimens had internal tissue protruding beyond the section edge, obscuring the cuticle. These specimens were discarded. In total, measurements of nine individuals from each of the pyrethroid intolerant and tolerant samples were obtained.

**Figure 4:**
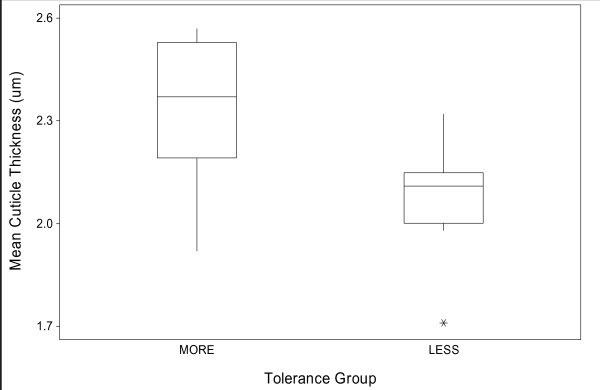
**Means and variation in cuticle thicknesses (with 95% limits) of two samples of *An. funestus *laboratory reared females characterised as either more or less tolerant to permethrin intoxication**.

The mean cuticle thickness of the intolerant specimens was 2.13 μm (SD ± 0.10 μm) while the tolerant specimens showed a mean thickness of 2.33 μm (SD ± 0.22 μm), giving a mean difference of 0.20 μm (Figure 4). This difference is significant based on ANOVA (P = 0.03).

**Figure 5:**
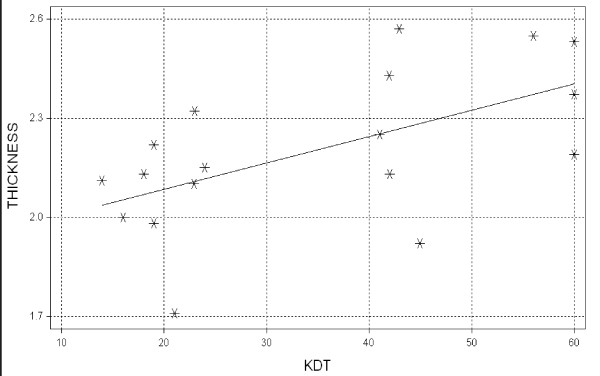
**Time-to-knockdown (KDT) during exposure to permethrin vs. mean cuticle thickness (microns)**. The trend is significant but weakly correlated (R^2 ^= 0.33, P = 0.03).

A linear regression of time to knock-down (*kd*_t_) vs. mean cuticle thickness is shown in Figure 5. There is a significant trend (P = 0.01) in which cuticle thickness generally increases with increasing length of time to knock-down, although the correlation is weak (R^2 ^= 0.33).

### Permethrin susceptibility assay

Measurements of mean cuticle thickness were obtained from 10 females characterised as 10-day-old permethrin resistant and 9 females characterised as 10-day-old permethrin susceptible. Only one specimen was discarded because of tissue obscuring the cuticle. There was no significant difference in wing-length measurements between the two samples (mean wing-length of resistant sample = 2.86 mm; mean wing-length of susceptible sample = 2.88 mm; P = 0.7084 based on a two sample t test).

**Figure 6:**
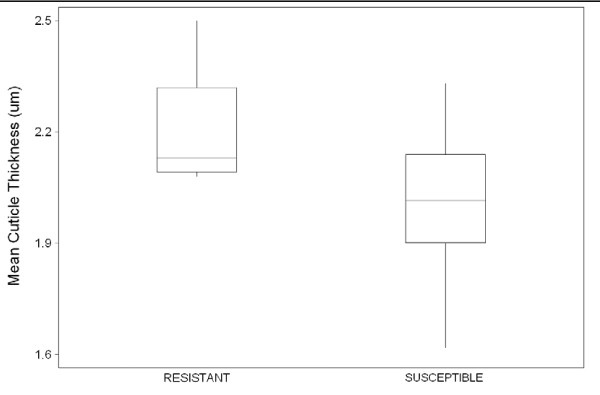
**Means and variation in cuticle thicknesses (with 95% limits) of two samples of *An. funestus *laboratory reared females characterised as either resistant or susceptible to permethrin intoxication**.

The mean cuticle thickness of the permethrin susceptible specimens was 2.00 μm (SD ± 0.20 μm) while the permethrin resistant specimens showed a mean thickness of 2.21 μm (SD ± 0.15 μm) (Figure 6), giving a mean difference of 0.21 μm. This difference is significant (P = 0.02) based on one-way ANOVA.

### Gender comparison

One male specimen was discarded owing to tissue obscuring the cuticle, and measurements were obtained from 11 specimens. Two female specimens were discarded and cuticle measurements were obtained from 10 specimens. There was no significant variation in wing length between samples (mean wing-length of males = 3.01 mm; mean wing-length of females = 3.12 mm; P = 0.07 based on a 2 sample t test).

**Figure 7:**
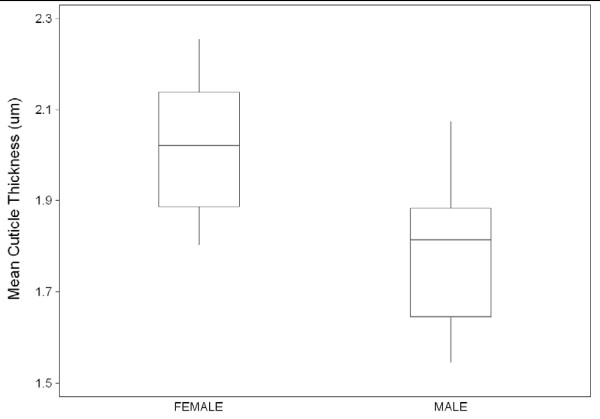
**Means and variation in cuticle thicknesses (with 95% limits) of female and male *An. funestus *samples drawn from a laboratory colony that is fully susceptible to insecticide**.

Mean cuticle thicknesses were 1.79 μm (SD ± 0.18 μm) for the male sample and 2.01 μm (SD ± 0.15 μm) for the female sample (Figure 7). The mean difference of 0.22 μm was significant based on ANOVA (P = 0.01).

## Discussion

Insecticide resistant phenotypes and their underlying mechanisms tend to evolve rapidly under intense insecticide selection pressure, leading to the prediction that resistance, in general, is likely to be conferred by a small number of major-effect genes [22]. However, differential expression of many genes not normally associated with insecticide resistance may also occur [23].

Measurements of response to permethrin exposure, expressed either in terms of tolerance during exposure or outcome following exposure, show similar associations with mean cuticle thickness. There was a 9.5-10% increase in mean cuticle thickness in those individuals either more tolerant or resistant to permethrin compared to those that were either less tolerant or permethrin susceptible. A link between cuticle thickness and response to insecticide exposure is thus based on the assortment of phenotypes, whereby the permethrin tolerant and resistant groups were significantly associated with thicker cuticles and vice versa. A similar difference in cuticle thickness was observed between insecticide susceptible males and females which may contribute to the differences in tolerance to insecticide intoxication generally observed between genders in *An. funestus *[8]. Variation in the expression of pyrethroid resistance with age in the FUMOZ colony is most likely based on variation in monooxygenase gene expression. Ten day old FUMOZ mosquitoes show reduced insecticide resistance compared to younger cohorts [8], but our data give no indication as to whether this variation is in any way linked to cuticle deposition.

These data support the hypothesis that the efficiency of monooxygenase-based pyrethroid resistance in southern African *An. funestus *is likely to be affected by minor factors. This is because in order to produce an effective resistant phenotype, upregulated transcription of selected P450 monooxygenase genes must produce sufficient enzyme to catalyze the metabolism of pyrethroids at a rate that prevents significant interaction between the insecticide and its neuronal target. Therefore, any mechanism that slows or regulates insecticide inoculation of internal organs, such as cuticular thickening, is a likely candidate for selection along with the primary mode of resistance.

## Conclusion

We conclude that pyrethroid tolerant or resistant *An. funestus *females are likely to have thicker cuticles than less tolerant or susceptible females, and that females generally have thicker cuticles than males. In pyrethroid resistant *An. funestus*, this increase in cuticle thickness is likely to have developed as an auxiliary to the primary mode of resistance which is based on enzyme-mediated detoxification.

Insecticide resistance has the potential to undermine insecticide based vector control applications. Resistance management strategies, which aim to either circumvent or reduce the rate of insecticide resistance development in vector populations, are best served by as complete an understanding of resistance mechanisms and their potential effects as possible.

## List of Abbreviations

SEM: Scanning Electron Microscopy; WHO: World Health Organisation; ANOVA: Analysis of Variance.

## Competing interests

The authors declare that they have no competing interests.

## Authors' contributions

ORW assisted with experimental design, conducted the experiments, analysed the data and drafted the initial manuscript. SH assisted with experimental design and data interpretation, offered general expertise and advice, and reviewed the manuscript prior to submission. MC assisted with data interpretation and reviewed the manuscript prior to submission. LLK assisted with experimental design and reviewed the manuscript prior to submission. BDB assisted with experimental design, data analysis and interpretation, and produced the final version of the manuscript.

## References

[B1] Hargreaves K Koekemoer LL Brooke BD Hunt RH Mthembu J Coetzee M *Anopheles funestus *resistant to pyrethroid insecticides in South Africa Med Vet Entomol 2000 14 181 189 10.1046/j.1365-2915.2000.00234.x 10872862

[B2] Brooke BD Kloke G Hunt RH Koekemoer LL Temu EA Taylor ME Small G Hemmingway J Coetzee M Biosassay and biochemical analyses of insecticide resistance in southern African *An. funestus *(Diptera: Culicidae) Bull Entomol Res 2001 91 265 272 11587622 10.1079/ber2001108

[B3] Coetzee M Knols BGJ, Louis C Malaria and dengue vector biology and control in southern and eastern Africa. Chapter 9 Bridging Laboratory and Field Research for Genetic Control of Disease Vectors 2005 Wageningen UR Frontis 101 109 Series #11

[B4] Maharaj R Mthembu DJ Sharp BL Impact of DDT re-introduction on malaria transmission in KwaZulu-Natal S Afr Med J 2005 95 871 874 16344885

[B5] Wondji CS Morgan J Coetzee M Hunt RH Steen K Black WC IV Hemingway J Ranson H Mapping a quantitative trait locus (QTL) conferring pyrethroid resistance in the African malaria vector *Anopheles funestus* BMC Genomics 2007 8 34 10.1186/1471-2164-8-5 17261170 PMC1790900

[B6] Wondji CS Irving H Morgan J Lobo NF Collins FH Hunt RH Coetzee M Hemingway J Ranson H Two duplicated P450 genes are associated with pyrethroid resistance in *Anopheles funestus*, a major malaria vector Genome Res 2009 19 452 459 10.1101/gr.087916.108 19196725 PMC2661802

[B7] Amenya DA Naguran R Lo T-C M Ranson H Spillings BL Wood OR Brooke BD Coetzee M Koekemoer LL Over expression of a cytochrome P450 (CYP6P9) in a major African malaria vector, *Anopheles funestus*, resistant to pyrethroids Insect Mol Biol 2008 17 19 25 18237281 10.1111/j.1365-2583.2008.00776.x

[B8] Hunt RH Brooke BD Pillay C Koekemoer LL Coetzee M Laboratory selection for and characteristics of pyrethroid resistance in the malaria vector *Anopheles funestus* Med Vet Entomol 2005 19 271 275 10.1111/j.1365-2915.2005.00574.x 16134975

[B9] Ahmad M Denholm I Bromilow RH Delayed cuticular penetration and enhanced metabolism of deltamethrin in pyrethroid-resistant strains of *Helicoverpa armigera *from China and Pakistan Pest Manag Sci 2006 62 805 810 10.1002/ps.1225 16649192

[B10] Gunning RV Devonshire AL Moores GD Metabolism of esfenvalerate by pyrethroid susceptible and resistant Australian *Helicoverpa armigera *(Lepidoptera: Noctuidae) Pestic Biochem Physiol 1995 51 205 213 10.1006/pest.1995.1020

[B11] Neville AC Circadian organization of chitin in some insect skeletons J Microscop Sci 1965 106 315 325

[B12] Dingle H Caldwell RL Haskell JB Temperature and circadian control of cuticle growth in the bug, *Oncopeltus fasciatus* J Insect Physiol 1969 15 373 378 10.1016/0022-1910(69)90284-4

[B13] Tyndale-Biscoe M Age-grading methods in adult insects: a review Bull Entomol Res 1984 74 341 377 10.1017/S0007485300015637

[B14] Cook PE Hugo LR Iturbe-Ormaetxe I Williams CR Chenoweth SF Ritchie SA Ryan PA Kay BH Blows MW O'Neill SL The use of transcriptional profiles to predict adult mosquito age under field conditions Proc Natl Acad Scis 2006 103 18060 18065 10.1073/pnas.0604875103 PMC1838706 17110448

[B15] Hanrahan SA Ageing of field and laboratory reared African migratory locusts by means of cuticle growth J Entomol Soc South Afr 1992 55 59 69

[B16] Pedrini N Mijailovsky SJ Girotti JR Stariolo R Cardozo RM Gentile A Juarez MP Control of pyrethroid-resistant Chagas disease vectors with entomopathogenic fungi PLoS Negl Trop Dis 2009 3 e434 10.1371/journal.pntd.0000434 19434231 PMC2674565

[B17] Vontas J David JP Nikou D Hemingway J Christophides GK Louis C Ranson H Transcriptional analysis of insecticide resistance in *Anopheles stephensi *using cross-species microarray hybridization Insect Mol Biol 2007 16 315 324 10.1111/j.1365-2583.2007.00728.x 17433071

[B18] David JP Strode C Vontas J Nikou D Vaughan A Pignatelli PM Louis C Hemingway J Ranson H The *Anopheles gambiae *detoxification chip: a highly specific microarray to study metabolic based insecticide resistance in malaria vectors Proc Natl Acad Scis 2005 102 4080 4084 10.1073/pnas.0409348102 PMC554807 15753317

[B19] Okoye P Biology of insecticide resistance in the African malaria vector *Anopheles funestus* 2008 PhD thesis University of the Witwatersrand Johannesburg, South Africa Animal, Plant and Environmental Sciences

[B20] World Health Organization Test procedures for insecticide resistance monitoring in malaria vectors bio-efficacy and persistence of insecticides on treated surfaces 1998 Geneva Switzerland Document *WHO/CDS/CPC/MAL/98.12*

[B21] Lyimo EO Takken W Effects of adult body size on fecundity and the pre-gravid rate of *Anopheles gambiae *females in Tanzania Med Vet Entomol 1993 7 328 332 10.1111/j.1365-2915.1993.tb00700.x 8268486

[B22] Ffrench-Constant RH Daborn PJ Le Goff G The genetics and genomics of insecticide resistance Trends in Genetics 2004 20 163 170 10.1016/j.tig.2004.01.003 15036810

[B23] Vontas J Bass C Koutsos AC David J-P Kafatos FC Louis C Hemmingway J Christophides GK Ranson H Gene expression in insecticide resistant and susceptible *Anopheles gambiae *strains constitutively or after insecticide exposure Insect Mol Biol 2005 14 509 521 10.1111/j.1365-2583.2005.00582.x 16164607

[B24] Evans AM Mosquitoes of the Ethiopian Region. II 1938 London: British Museum (Natural History)

